# The correlation between insulin-like growth factor binding protein 1 (IGFBP-1) and homeostasis model assessment of insulin resistance (HOMA-IR) in polycystic ovarian syndrome with insulin resistance

**Published:** 2018-11

**Authors:** Arini Firmansyah, Maisuri Tadjuddin Chalid, Retno Budiati Farid, Nusratuddin Nusratuddin

**Affiliations:** *Department of Obstetrics and Gynecology Faculty of Medicine Universitas Hasanuddin Makassar, Indonesia.*

**Keywords:** Insulin-like growth factor binding protein 1, Insulin resistance, Polycystic ovarian syndrome

## Abstract

**Background:**

The underlying etiology of polycystic ovarian syndrome (PCOS) is unknown and assumed to have a strong correlation with insulin resistance. Homeostasis Model Assessment of insulin resistance (HOMA-IR) is a good tool to assess insulin resistance. Low levels of serum Insulin-like Growth Factor-Binding Protein-1 (IGFBP-1) in PCOS women led to the hypothesis that hyperinsulinemia in PCOS inhibits the production of IGFBP-1, which in turn stimulates excessive androgen production.

**Objective:**

The study is aimed to analyze the correlation between the levels of IGFBP-1 and HOMA-IR on insulin resistance in PCOS.

**Materials and Methods:**

A cross-sectional study among 105 PCOS women, including 60 women with insulin resistance were recruited. The mean of IGFBP-1 and HOMA-IR were 6.507±4.7821 μg/l and 3.633±1.666 respectively.

**Results:**

Low levels of IGFBP-1 were detected in all insulin resistance women. There was a correlation between HOMA-IR and overweight (p=0.045), while IGFBP-1 showed no correlation with overweight (p=0.106). In addition, no correlation between IGFBP-1 with HOMA-IR as a marker of insulin resistance was detected.

**Conclusion:**

Despite the decrease in IGFBP-1, it seems that there is no correlation between IGFBP-1 with HOMA-IR as a marker of insulin resistance.

## Introduction

Polycystic ovarian syndrome (PCOS) is the most common endocrinopathy disorder in women of reproductive age with an incidence of 4-12%. PCOS is characterized by the accumulation of undeveloped follicles in the ovaries associated with ovarian androgen production. In its complete form, this syndrome gives an overview of polycystic ovary, amenorrhea and hirsutism (1). The etiology underlying PCOS is unknown. Current studies were focusing on the role of insulin secretion and action in the pathophysiology of PCOS, which was assumed to have a strong correlation with insulin resistance. As many as 70% of women with PCOS have insulin resistance (IR) (2). Insulin resistance is a condition where fat, muscle, and liver have a lowered response to endogenous or exogenous insulin (3).

In women with PCOS, insulin resistance with compensatory hyperinsulinemia may induce ovarian androgen overproduction resulting in hyperandrogenism. Insulin works on androgen production in the ovaries through type I insulin-like growth factor (IGF-1) receptors in both theca and stromal cells (4). Both in normal human ovaries and polycystic ovaries, granulosa cells can secrete insulin-like growth factor-binding protein-1 (IGFBP-1) in response to follicular stimulating hormone (5). IGFBP-1 is part of the IGFBP family and encodes proteins with IGFBP domains. IGFBP-1 is found in serum. In normal individuals, IGFBP-1 concentrations fluctuate up to 10 times as a response to acute changes in insulin levels that lead to the inhibition of the IGFBP-1 transcription gene and its production in the liver. The mean value of IGFBP-1 in a normal individual was 26.1-124.0 μg/l. The low serum levels of IGFBP-1 in PCOS women led to the hypothesis that insulin resistance in PCOS inhibits the production of IGFBP-1 which in turn stimulates excess androgen production in PCOS. 

Homeostasis Model Assessment of Insulin Resistance (HOMA-IR) is a good tool to assess insulin resistance in vivo and the precision is as good as the glucose clamp technique (6). Furthermore, HOMA-IR can be used as an easy and sensitive clinical index to assess ovarian function in PCOS women with insulin resistance (2). There is still no conclusion about the cut off point used in the diagnosis of insulin resistance. Matthews and colleagues stated HOMA-IR level over 2.5 for the diagnosis of insulin resistance in the general population whereas Jensterle and colleagues used a minimal HOMA-IR value of 2 (7). 

This study aims to analyze the correlation between the levels of IGFBP-1 and HOMA-IR in insulin resistance PCOS.

## Materials and methods

A cross-sectional study took place from January to December 2016 at Wahidin Sudirohusido Hospital, Makassar, Indonesia. We used the Rotterdam 2003 criteria for selection of the patients as 2 out of 3 criteria: 1. oligo-and/or anovulation, 2. clinical and/or biochemical signs of hyperandrogenism and 3. polycystic ovaries (3). The inculsion criteria were as followed: female age 18-40 years, hyperinsulinemia explained by HOMA-IR >2.00, and BMI 18.5-35. 

A total of 105 women were diagnosed with PCOS, 60 of them were considered eligible for enrolment and undergo IGFBP-1 examination. The exclusion criteria were as followed: Pregnant or lactating, Cushing syndrome, adrenal congenital hyperplasia, diabetes mellitus, acute or chronic infection, malignancy, on weight loss program, on laparoscopic ovarian diathermy, on in vitro fertilization program, and on medication therapy that may affect insulin sensitivity. 

HOMA-IR was calculated as: fasting insulin (µu/ml)×fasting glucose (mmol/lit) /22.5. Six ml of venous blood sample was taken for fasting blood glucose (FPG) examination and was proceeded for HOMA-IR measurement if FPG <126 mg/dL. The blood sample then undergo further IGFBP-1 analysis using enzyme-linked immunosorbent Assay (ELISA) kit of Bt Laboratory. Of the entire sample, overweight and non-overweight criteria were classified according to by body mass index (BMI). BMI is a person's weight in kilograms (kg) divided by his or her height in meters squared (m^2^). In this study, we classified BMI ≤25 kg/m^2 ^as non-overweight group, and BMI 25-29.9 kg/m^2 ^as overweight group.

**Ethical consideration**

All procedures were approved by the Ethics Committee of Medical Research, Faculty of Medicine, Hasanuddin University in Indonesia (Approval letter IRB protocol No: UH1601004). Of all the women who met the diagnostic criteria, the subjects gave their written consent before the start of any procedure.

**Statistical analysis**

SPSS software (Statistical Package for the Social Sciences, version 22.0, SPSS Inc, Chicago, Illinois, USA) was used for data analysis. The Mann-Whitney and Spearman's rho correlation tests were used to report the associations between variables, and p<0.05 was considered statistically significant.

## Results

Results from table I show the main sample characteristics between 20-29 yr, infertile and overweight with abdominal circumference>80 cm. According to WHO protocol, measuring of WC is made at the midpoint of the lowest rib and at the top of iliac crest (8). The mean waist circumference for non-overweight is 88.07±3.23. A waist circumference value more than 88 cm is an indicator for abdominal obesity (8). Despite the insulin resistance, no hyperinsulinemia was found in all samples. All sample shows normal FPI with range below 28.5 uIU/ml (Table II). 

**Figure 1:**
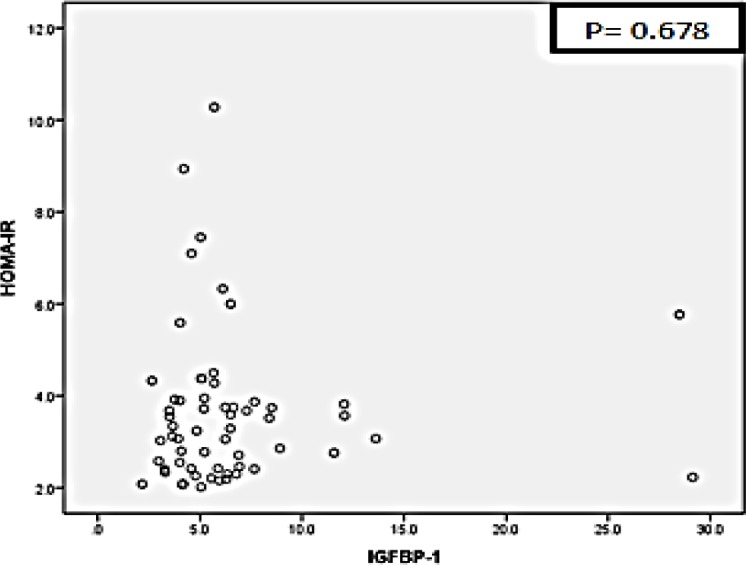
Scattered data distribution of IGFBP-1 with HOMA-IR. p=0.678 (Spearman’s rho test)

PCOS patients were dominated by clinical characteristics of overweight and infertility (Table I). All samples showed normal FPG despite a slight increase of 2 hr post prandial plasma glucose (Table II). The mean and standard deviation of IGFBP-1 levels were 6.507±4.7821, and HOMA-IR levels were 3.633±1.6662. Statistic test results using Spearman's rho correlation test shows that there was no correlation between IGFBP-1 levels and insulin resistance using HOMA-IR parameter (p=0.678) (Figure 1).

The average HOMA-IR (3.873±1.8208) in the overweight group subjects was higher than this (3.025±0.9930) in the non-overweight group. Simillarly, the average levels of IGFBP-1 in the obese group (7.118±5.4451) was higher than this (4.962±1.7203) in the non-obese subjects. Based on statistic test results using Mann-Whitney test there was a correlation between HOMA-IR and obesity (p=0.045), while IGFBP-1 showed no correlation with obesity (p=0.106) (Table III).

**Table I:** Subject characteristic

**Characteristic**		**Outcome**
Age (yr)		
	20-29	34 (56.7)
	30-39	26 (43.3)
Infertility		
	Infertile	56 (93.3)
	Non infertile	4 (6.7)
BMI		
	>25	43 (71.7)
	<25	17 (28.3)
Waist Circumference		
	>80 cm	57 (95.0)
	<80 cm	3 (5.0)

Data presented as n(%).

BMI= Body Mass Index

**Table II:** Subject characteristic based on Body Mass Index (BMI)

**Characteristic**	**Non-overweight**	**Overweight**
Age (yr)	28.4 ± 2.120	29.5 ± 3.978
WC (cm)	88.07 ± 3.23	89.7 ± 4.538
FPG (mg/dL)	94.68 ± 8.366	89.65 ± 14.463
2HPP (mg/dL)	148.28 ± 31.315	147.12 ± 34.191
FPI (uIU/mL)	15.44 ± 3.623	13.58 ± 7.351

Data presented as mean ± SD.

WC= Waist circumference

FPG= Fasting plasma glucose

2HPP= 2 hr post prandial plasma glucose

FPI= Fasting plasma insulin

**Table III:** Analysis of HOMA-IR and IGFBP-1 Levels among overweight and non-overweight PCOS

**Variable**		**Number (n=60)**	**Mean**	**p-value**
HOMA-IR level				
	Overweight	43	3.873 ± 1.8208	0.045
	Non-overweight	17	3.025 ± 0.9930	
IGFBP-1 level				
	Overweight	43	7.118 ± 5.4451	0.106
	Non-overweight	17	4.962 ± 1.7203	

Mann-whitney test

HOMA-IR: Homeostasis model assessment of insulin resistance

IGFBP-1: Insulin-like growth factor-binding protein-1

BMI= Body mass index

## Discussion

The mechanism of PCOS caused by insulin resistance and hyperinsulinemia remains unexplained. One theory suggests that increased serum insulin concentrations have a direct action in increasing androgen production via IGF-1 receptors (9), but these receptors have a higher affinity for IGF-1 than insulin. Both in healthy and in diabetics individuals, insulin administration shows a decrease in serum IGFBP-1 (10).

There is a particular binding site for both insulin and Insulin Growth Factor-1 (IGF-1) in a healthy ovarian stroma. Both insulin and IGF-1 potentiate androstenedione and luteinizing hormone-induced testosterone secretion in cultured human theca cells (11). Only dominant follicle’s granulosa cells shows a prevalent expression of IGFBP-1’s mRNA (6). Furthermore, studies have shown that both insulin and IGF-1 may inhibit IGFBP-1 production through insulin and IGF receptors (11). These findings indicate that the decrease in IGFBP-1 produced by granulosa cells is less likely to act as an IGF-I bioactivity antagonist in stromal-cell resulting in androgen overproduction and in turn leading to follicular and anovulatory maturation defect (12). 

In this study all samples of PCOS women with insulin resistance were measured with HOMA-IR levels, it was found that the mean IGFBP-1 level was 6.507 μg/l ±4.782 SD. This value was similar to a range of a systematic review and meta-analysis results which describe that the mean IGFBP-1 levels in the PCOS population were 6.4 μg/l to 26.9 μg/l (8). The IGFBP-1 levels in this study were also consistent with the findings of Morris and Falcon who compared the value with a control group and found that IGFBP-1 levels in the PCOS were lower (13). Homa-IR levels in this study were 3.63±1.666. This clearly indicates a condition of insulin resistance in the study samples. A study published in the Journal of Nutrition and Metabolism exposes that the optimal Homa-IR cut off value in diagnosing insulin resistance in non-diabetic patients is 1.775 (14).

Using bivariate analysis (Table III), we did not find any correlation between IGFBP-1 concentration and insulin resistance using HOMA-IR parameter. This result indicates that although IGFBP-1 levels in PCOS tend to be low, there was no involvement of IGFBP-1 in PCOS mechanisms with insulin resistance. This was due to the absence of hyperinsulinemia samples despite the overall sample of insulin resistance. Consistently low levels of IGFBP-1 in all samples indicate the possibility of other pathways involved in the pathogenesis of low IGFBP-1 levels in PCOS. This finding was in line with Kelly's conclusion that IGFBP-1 did not play a major role in the pathogenesis of PCOS (10). Further investigation of other factors affecting IGFBP-1 interactions and insulin resistance is required, in this case, IGF-1. Insulin resistance in PCOS women increases the levels of free bioavailability of IGF-1 and may contribute to abnormalities in ovarian steroidogenesis. Abnormalities of ovarian steroidogenesis and insulin resistance cause a chronic positive feedback loop that worsens PCOS. In addition, IGF-1 also has a role in inhibiting the production of IGFBP-1 and causing low IGBP-1 levels in PCOS

## Conclusion

IGFBP-1 values decreased in all PCOS women in the study. Despite the decrease in IGFBP-1, it has no correlation with HOMA-IR as a marker of resistance. Moreover, when considering insulin resistance in PCOS, further evaluation on the interaction between IGFBP-1 and IGF-1 is required for a better understanding of the pathophysiology.

## References

[1] Cunningham F, Schorge G, Cunningham (2008). Polycystic Ovarian Syndrome, and Hyperandrogenism. Williams Gynecology.

[2] Traub ML (2011). Assessing and treating insulin resistance in women with polycystic ovarian syndrome. World J Diabetes.

[3] Fritz MA & Speroff L, Seigafuse (2011). Chronic anovulation and polycystic ovary syndrome. Clinical gynecologic endocrinology and infertility.

[4] Nagamani M, Stuart CA (1990). Specific binding sites for insulin-like growth factor I in the ovarian stroma of women with polycystic ovarian disease and stromal hyperthecosis. Am J Obstet Gynecol.

[5] Bergh C, Carlsson B, Olsson JH, Selleskog U, Hillensjo T (1993). Regulation of androgen production in cultured human thecal cells by insulin-like growth factor I and insulin. Fertil Steril.

[6] Gayoso-Diz P, Otero-González A, Rodriguez-Alvarez MX, Gude F, García F, De Francisco A (2013). Insulin resistance (HOMA-IR) cut-off values and the metabolic syndrome in a general adult population: effect of gender and age: EPIRCE cross-sectional study. BMC Endoc Disord.

[7] Wongwanaruk T, Rattanachaiyanont M, Leerasiri P, Indhavivadhana S, Techatraisak K, Angsuwathan S (2012). The usefulness of homeostatic measurement assessment-insulin resistance (HOMA-IR) for detection of glucose intolerance in thai women of reproductive age with polycystic ovary syndrome. Int J Endocrinol.

[8] (2008). Waist circumference and waist-hip ratio: Report of a WHO expert consultation.

[9] Kelly CJ, Stenton SR, Lashen H (2011). Insulin-like growth factor binding protein-1 in PCOS: a systematic review and meta-analysis. Hum Reprod Update.

[10] Barbieri RL, Rayan KJ (1983). Hyperandrogenism, insulin resistance and acanthosis nigricans syndrome: a common endocrinopathy with distinct pathophysiologic features. Am J Obstet Gynecol.

[11] Barbieri RL (1994). Hyperandrogenism, insulin resistance and acanthosis nigrican 10 years of progress. J Reprod Med.

[12] Mason HD, Margara R, Winston RM, Seppala M, Koistinen R, Franks S (1993). Insulin-like growth factor-I (IGFI) inhibits production of IGF-binding protein-1 while stimulating estradiol secretion in granulosa cells from normal and polycystic human ovaries. J Clin Endocrinol Metab.

[13] Morris DV, Falcon T (1996). The relationship between insulin sensitivity and Insulin-like growth factor-binding protein-1. Gynecol Endocrinol.

[14] Esteghamati A, Ashraf H, Khalilzadeh O, Zandieh A, Nakhjavani M, Rashidi A (2010). Optimal cut-off of homeostasis model assessment of insulin resistance (HOMA-IR) for the diagnosis of metabolic syndrome: third national surveillance of risk factors of noncommunicable diseases in Iran (SuRFNCD-2007). Nutr Metab (Lond).

